# Two-stage hemoglobin prediction based on prior causality

**DOI:** 10.3389/fpubh.2022.1079389

**Published:** 2022-11-30

**Authors:** Yuwen Chen, Kunhua Zhong, Yiziting Zhu, Qilong Sun

**Affiliations:** ^1^Chongqing Institute of Green and Intelligent Technology, Chinese Academy of Sciences, Chongqing, China; ^2^Department of Anesthesiology, The First Affiliated Hospital of Chongqing Medical University, Chongqing, China

**Keywords:** non-invasive, prediction, causal knowledge, hemoglobin, segmentation

## Abstract

**Introduction:**

Perioperative hemoglobin (Hb) levels can influence tissue metabolism. For clinical physicians, precise Hb concentration greatly contributes to intraoperative blood transfusion. The reduction in Hb during an operation weakens blood's oxygen-carrying capacity and poses threats to multiple systems and organs of the whole body. Patients can die from perioperative anemia. Thus, a timely and accurate non-invasive prediction for patients' Hb content is of enormous significance.

**Method:**

In this study, targeted toward the palpebral conjunctiva images in perioperative patients, a non-invasive model for predicting Hb levels is constructed by means of deep neural semantic segmentation and a convolutional network based on a priori causal knowledge, then an automatic framework was proposed to predict the precise concentration value of Hb. Specifically, according to a priori causal knowledge, the palpebral region was positioned first, and patients' Hb concentration was subjected to regression prediction using a neural network. The model proposed in this study was experimented on using actual medical datasets.

**Results:**

The *R*^2^ of the model proposed can reach 0.512, the explained variance score can reach 0.535, and the mean absolute error is 1.521.

**Discussion:**

In this study, we proposed to predict the accurate hemoglobin concentration and finally constructed a model using the deep learning method to predict eyelid Hb of perioperative patients based on the a priori casual knowledge.

## Introduction

Hemoglobin (Hb) is a protein mainly responsible for transporting oxygen in higher organisms. Hb as a pivotal participant in multiple life activities of organisms is capable of effectively delivering oxygen, transferring electrons, and promoting the decomposition of hydrogen peroxide. In clinical practice, illnesses such as anemia, leukemia, and cardiac diseases are usually associated with Hb disorders. For instance, anemia is a disease syndrome involving multiple systems, which may be attributed to the reduction in erythrocyte or Hb production, functional iron deficiency (e.g., sufficient iron stores or insufficient iron mobilization), and immune activation or suppression of erythrocyte production. Anemia has an incidence rate of about 25 percent worldwide (1), and notably increased cases are found in economically disadvantaged areas and in patients with malignant tumors. Mounting data have indicated the significant correlations of the incidence and fatality of perioperative complications in patients with anemia (2). Early interventions on patients with anemia can avoid organ dysfunction, improve nutritional status (3), correct anemia-induced cerebral cell ischemia and edema (4–6), cause a lower prosthesis-related infection rate after artificial replacement (7), lead to an incidence rate of cardiopulmonary bypass-induced renal damage (8), and reduce the occurrence of eclampsia and preeclampsia during pregnancy (9). Consequently, Hb content in human blood is considered as an important indicator to be watched during perioperative period.

Palpebral conjunctiva is a mucous membrane with superficial and rich capillaries not readily influenced by skin color and temperature. The color of palpebral conjunctiva tends to be gradually lighter with the reduction in Hb concentration and erythrocytes. Given that the deep or light color of palpebral conjunctiva reflects the anemic degree, a visual method is commonly used to rapidly assess the anemia of patients, though large differences exist among different evaluators (10, 11). The colorimetric card method is recommended by WHO to directly estimate Hb concentration after blood sample processing (12), which is superior to the visual method in terms of accuracy, but it is also limited due to invasive procedures and subjective assessment. Non-invasive pulse Hb detection (SpHb) based on a multi-wavelength spectrum provides real-time monitoring of Hb changes (13), which has been applied in some medical institutions. However, its detection accuracy is susceptible to finger temperature and posture changes (14), and specific instruments and expensive costs are needed. Thus, such a method has not been popularized. In addition, numerous non-invasive technologies and tools can indirectly determine the Hb level in blood and the oxygen content in human tissues, such as photoplethysmography (PPG) based on the oral mucosa and conjunctival tissues (15), reflectance spectroscopy of finger and palpebral conjunctiva (16), and fluorescence spectroscopy (17), which are also reported in some studies of anemia prediction. However, many products are usually not available as portable or wearable devices, and their prices are unaffordable to some.

As artificial intelligence technology develops, image recognition and analysis based on computer vision technology have been widely applied in the medical field. Machine learning with recognition and analysis of palpebral conjunctival images has a higher accuracy than physicians' visual method and overcomes the shortcomings resulting from insufficient medical staff as well as lack and backwardness of medical equipment. Moreover, a blood transfusion is needed for anemic patients in accordance with Hb levels, which should be monitored frequently. Although Hb levels are usually detected in an invasive manner, invasive procedures are not recommended for specific populations such as infants, elderly people, pregnant women, anemic patients, and those with sickle cell disease. In addition, patients feel severe discomfort with frequent blood sampling, and the cost is fairly expensive, especially in areas with limited economic resources. Herein, exploring non-invasive approaches and designing corresponding tools to monitor the concentration of Hb are vital to reduce the cost to patients. Additionally, classification research with normal or anemia populations as research objects constitutes the majority of existing studies on palpebral conjunctival Hb, which has a small sample size and certain requirements for photography. However, with respect to a non-invasive prediction for patients' Hb during the perioperative period, the difficulties that need to be overcome include low pixel of camera equipment, changeable postures, insufficient exposure, and short photography duration (due to being unable to focus on eyes precisely). Thus, based on a priori causal knowledge, the present study adopted feature engineering and deep neural network methods to construct a model for predicting the specific value of palpebral conjunctival Hb concentration to provide a novel approach for real-time prediction of Hb in patients undergoing surgery through palpebral conjunctiva.

The main contributions of this paper are: First, we constructed an eyelid data set of surgical patients. Secondly, we propose a two-stage non-invasive hemoglobin concentration accurate prediction method based on deep neural semantic segmentation, deep convolution neural network and label distribution learning. Finally, we carried out experimental verification on the constructed eyelid data set.

The organizational structure of this paper is as follows: the first section briefly introduces the significance of hemoglobin prediction, and the second section introduces the related work. The third section introduces the model and method proposed in this paper; The fourth section introduces the relevant experiments and conclusions, and finally the full text is summarized.

## Related work

Deep convolutional neural network (CNN) has achieved great success in segmentation of medical images (MRI, CT, X-ray, etc.) and auxiliary diagnosis due to its excellent feature expression ability. Cernazanu-Glavan (18) proposed a method for bone structure segmentation in X-ray images using convolutional neural network. Giusti (19) proposed a fast medical image scanning method based on maximum pooling convolutional neural network. Literature (20, 21) proposed the method of realizing automatic cell membrane segmentation of electron microscopic image based on convolutional neural network. Non-invasive hemoglobin prediction based on deep convolutional neural network has been studied in recent years. The main work is to classify patients based on conjunctiva images for anemia. In terms of the feature engineering method based on palpebral conjunctival images, Shaun et al. (22) extracted R and G channel color features of eyelid conjunctiva and calculated erythema index (EI) to predict anemia, with accuracy of 70 and 72%, higher than the evaluation of three clinicians (60/57/64%), revealing strong correlations between EI and Hb. Suner et al. (23) photographed eyes with a digital camera of which palpebral conjunctiva was manually clipped, and R, G, and B features in the RGB color space of photographs were extracted through MATLAB software analysis, followed by associating the results with Hb concentration. The findings suggest the prediction accuracy, sensitivity, and specificity of 71, 69, and 72% respectively. Sevani (24) extracted RGB features of images, calculated the centroid of R, G and B of eyelid conjunctiva images based on k-means algorithm, and clustered them for anemia classification study, involving 36 training sets and 10 test sets, with an experimental accuracy of 90%. Anggraeni et al. (25) constructed a prediction model with a sample size of 20. The RGB spatial model features were extracted and then fitted into a linear regression equation, indicating an association of 92% between anemia and color features. However, the sample size for deriving the model was too small, and the generalization ability was weak. Tamir et al. (26) studied 19 eyelid conjunctival photos, which were obtained using their own Android application under appropriate lighting conditions. The red and green intensity values in the spectrum are compared by Sobel edge detection method. The results show that the best average intensity threshold difference between the two is 1.5, the average intensity difference between red and green in patients with anemia is < 1.5, while the average intensity difference between red and green in patients without anemia is more than 1.5. The accuracy of this method in predicting anemia was 78.9%. Chen et al. (27) amended the Kalman filter from the original linear penalty regression to non-linear penalty regression, extracted the R component from the RGB color model of 100 eyelid conjunctival images, and set the HB threshold to 11 g/dl. The experiment confirmed that the introduced Kalman filter can reduce the quantity of suspicious anemia.

In the current research field, a head-mounted camera connected to a mobile phone was first used by Vitoantonio et al. (28) to achieve a light balance between photographs, which was reported in a study with a cohort of 77 subjects (9 anemic patients and 68 healthy people). This study was performed with components a and b in a CIE color space and a component G in RGB as features to be extracted and to support vector machine (SVM) as a classifier. The results were indicative of a moderate correlation (49%) between image color features and an Hb value (49%), and the accuracy, specificity, and sensitivity were 84.4, 82.4, and 100%, respectively in predicting anemia. Subsequently, the camera equipment was improved into a portable and low-cost macro camera by the same team. The Hbmeter software was adopted to analyze photographs, and the following data were set: Hb < 10.5 g/dL: highly suspected anemia, 10.5 g/dL < Hb < 11.5 g/dL: suspected anemia, and Hb > 11.5 g/dL: low probability of anemia. RGB and CIELab features were extracted, a k neighbor classifier was fitted, and the model performance was tested by 10-fold cross-validation. The correlation index between component an in-color model, and Hb was analyzed in the study; the results indicated that the following inclusion of all samples (sample size of 113) and the correlation index between a and Hb was 0.726. After the conditions were defined (i.e., 28 < L < 82 and 16 < R = G = B < 233), partial pixels (either too dark or too bright) were filtered out, and the correlation index between a and Hb was 0.745. Based on the deep learning method, the R. Muthalagu team (29) selected mean color features in HIS space extracted from 127 eye pictures as the input layer to construct a three-layer artificial neural network (ANN), which achieved a satisfactory result in terms of predicting anemia (i.e., the sensitivity, specificity, and accuracy were 77.27, 96.11, and 91.3% respectively). Compared to the EI algorithm recommended by Collings et al. (sensitivity 74%, specificity 77%), the model performance has been notably enhanced. In the team of Chen (27), a classification study with a sample size of 100 was designed to evaluate the prediction model using a fast algorithm based on Markov distance (minimum distance classifier) and a robust algorithm based on SVM and ANN. The former extracted the high-tone pixel features of HIS and the intermediate pixel data features of RGB, while the latter contained 18 features (involving vascular texture feature), improved the HIS features in the fast algorithm, and finally assessed the model through sensitivity, specificity, and the Kappa index. The fast algorithm, SVM, and ANN had a sensitivity of 62, 78, and 75% and a specificity of 90, 83, and 83% respectively. Although SVM is better than ANN, the advantage of ANN has not been exhibited due to small sample size. Jain et al. (30), 99 original photographs were transformed into 3,103 photographs *via* enhancement technology, and favorable results (i.e., accuracy 97%, sensitivity 99% and specificity 95%) were obtained through extracting R and G components followed by the introduction of the ANN algorithm in prediction stage. This paper provides evidence to suggest that the data-driven deep learning algorithm introduced is able to enhance model performance prominently. However, although improvement is achieved through enhancing 99 original photographs by about 30 times, the training time is also increased, which can easily cause over-fitting rather than real enhancement in the generalization ability. In addition, a fully in-depth learning model with a sample size of 300 investigated by Bryan et al. (31) revealed that through introducing a 35-layer convolutional neural network (CNN) algorithm in the region of interest (ROI) detection stage, superimposing a seven-layer CNN algorithm in the process of clipping palpebral conjunctival images, and finally mapping features learned by computer of the function of neural network regression with the cut-off value of Hb as 11 g/dL, an optimal sensitivity (77%) was obtained, whereas the accuracy and specificity were only 42 and 36% respectively. Considering that the results of preliminary trials are not satisfactory, research samples are still being collected, which aims to further enhance calculation results by expanding sample sizes.

### Eyelid region location based on prior causal knowledge and mask RCNN

In this study, Y ϵ [6,18] was defined to represent the range of Hb content in patients, and I was set as the discrete space of the patient's

Through the past researches, the challenges of hemoglobin prediction include acquisition equipment, ways of image extraction, algorithm selection and accurate prediction value. And our study aims to propose a two-stage non-invasive hemoglobin concentration accurate prediction method based on deep learning algorithm. Then we implemented the verification on the constructed eyelid data set to show the performance of the model.

## Models and methods

**Figure 1:**
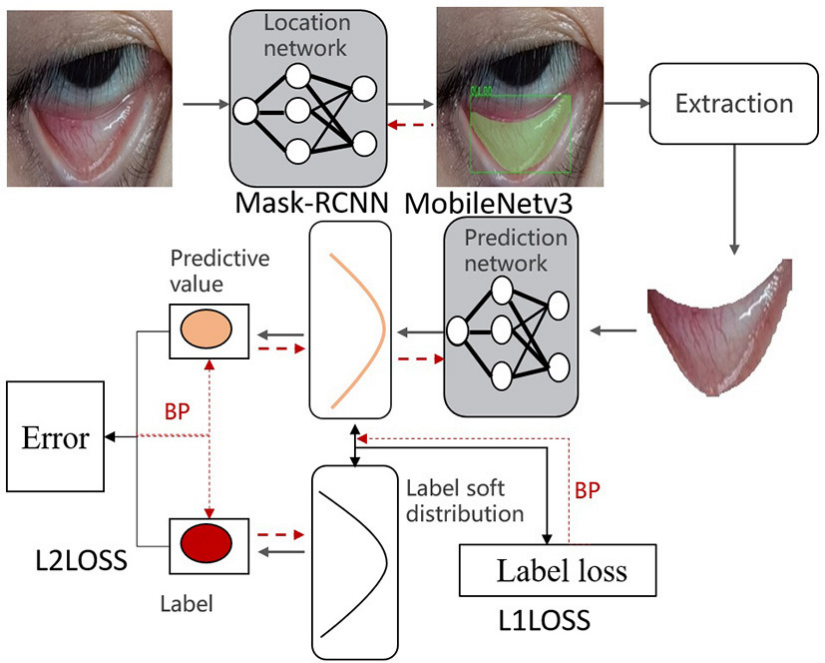
Architecture diagram of Hb concentration identification.

**Figure 2:**
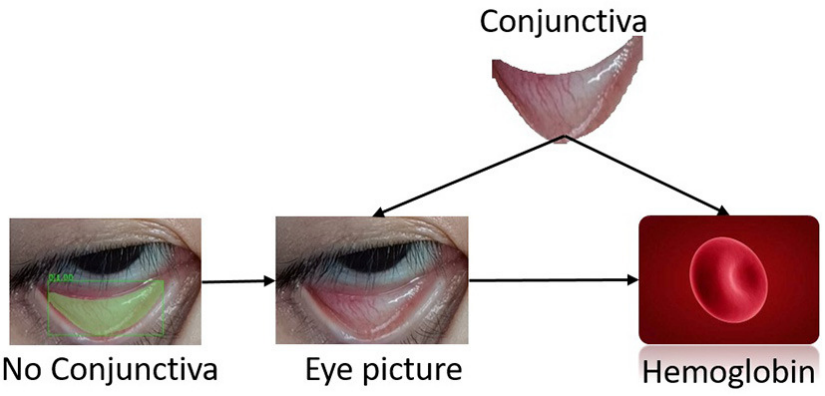
Cause-and-effect diagram of patient's Hb concentration.

Targeted toward perioperative patients' eye photographs taken by the mobile device, this study proposed an automatic framework to predict Hb concentration precisely (Figure 1). The first was to locate the palpebral region network, that is, the palpebral region was identified from an original eye photograph through a deep neural network. Then Hb concentration was subjected to a regression prediction based on a concentration prediction neural network. The details are presented as follows.

palpebral image. As shown in Figure 2, the intervention on the palpebral region in the image may influence the distribution of the Hb value Y. Thus, the function C: I → Y could be called the causal feature or visual cause of image I.


(1) $$C\left(I\right)\;=\;\left\{ \begin{array}{l} Y\;Containing\;palpebral\;pixels\\ \;0\;Else \end{array}\right.$$

Except for the palpebral region, there was no causality in the other parts (i.e., eyes, eyelashes, pupils, or skin), and an intervention on these pixels in the image had no impact on the prediction of the Hb value. However, the existence of these parts (eyes, eyelashes, pupils, and skin) was closely associated with the Hb value of patients (due to the common cause, i.e., palpebra). The following function S: I → Y was called as the false correlation of Y in image I:


(2) $$S\left(I\right)\;=\;\{\begin{array}{c} Y\;Containing\;pixels\;other\;than\;the\;palpebrae\\ 0\;Otherwise \end{array}$$

Both palpebral and non-palpebral pixels (e.g., eyes, eyelashes, pupils and skin) were predictors of the target variable (the patient's Hb value), but only one of them was the cause. For this reason, there was a need to distinguish the related factors (i.e., those with actual causality) of the target variable, so as to identify the visual cause from images.

In this study, the palpebra in the image was a causal factor predicting the Hb value of patients. The image implicated in classification labels was observed in the standard classification task of machine learning so the target variable could be predicted through images directly. However, the prediction probability of the image was not able to display the causality of the image to the target variable Y. Furthermore, Hb content was only correlated with the palpebral region of patients, so other noise information was introduced to facilitate data acquisition. Identifying the causal factors regarding prediction results contributed to enhancing the reliability and interpretability of the prediction. Consequently, for this study, based on the deep neural semantic segmentation network, the palpebral region was extracted by clipping the original palpebral photographs collected to realize the extraction of predictive causal features.

Mask RCNN (32), a highly efficient instance semantic segmentation model, was capable of realizing pixel-level image instance segmentation. The region of interest alignment algorithm (ROIAlign) and fully convolutional network (FCN) were designed on the basis of the (33). The semantic segmentation prediction and classification prediction were divided into two branches of the network. Specifically, the classification prediction branch provided a prediction for the ROIAlign and generated category labels and rectangular boxes, while the semantic segmentation prediction branch generated a binary mask of the image. In this study, the palpebral conjunctival region was first extracted on the basis of such highly-efficient and accurate segmentation of the neural network.

**Figure 3:**
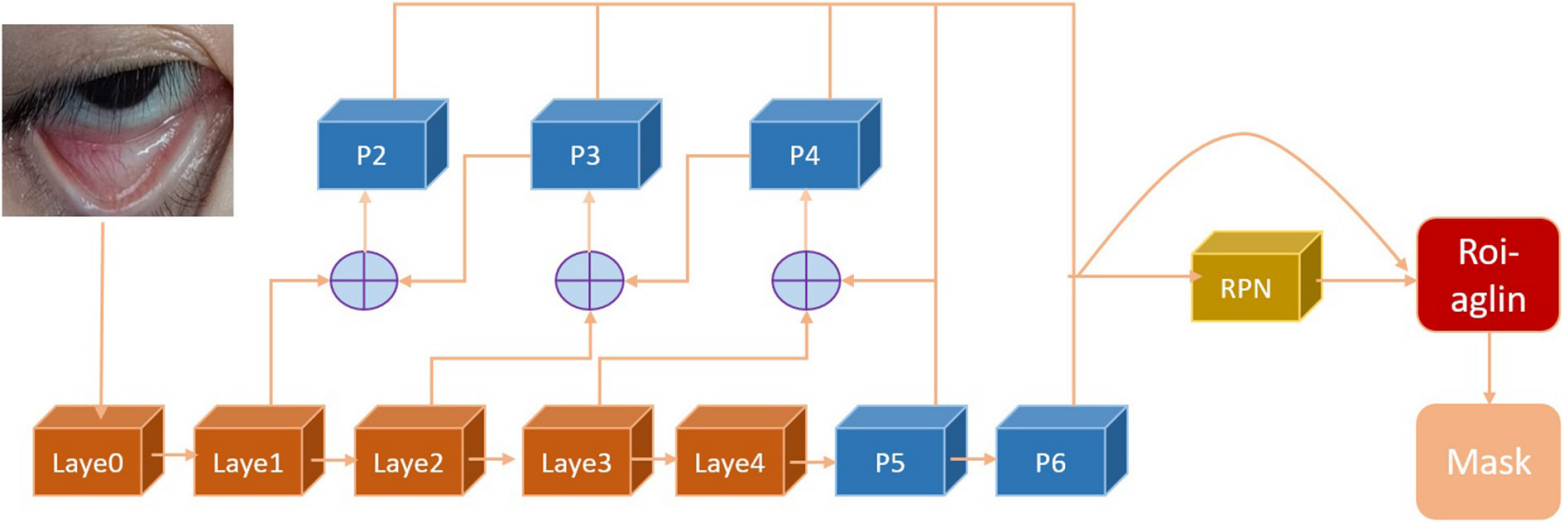
Flow chart of palpebral segmentation network.

As shown in Figure 3, an image involving the palpebral conjunctival region was input. Then forward reasoning was conducted through the palpebral positioning network to output the palpebral region of this image. Layer0, layer1, layer2, layer3, and layer4 were all blocks composed of network structures such as the convolutional layer, batch normal layer, and activation function. The FPN network constituted by p2, p3, p4, p5, and p6 consisted of a convolutional layer and up-sampling operation. The region proposal network (RPN) was mainly responsible for extracting the ROI from the image and generating the target candidate region. The combination of the ROI Align layer with the FPN feature layer and RPN layer to form a fixed feature layer contributed to the network calculation. The eye image to be processed is first inputted into the pre-trained ResNet+ FPN network to extract features and obtain the corresponding feature maps. The feature map obtains a considerable number of candidate frames (regions of interest or ROI) through RPN. Then, binary classification of the foreground and background is performed using the SoftMax classifier. More accurate candidate frame position information is obtained from frame regression. Additionally, part of the ROI is filtered out under non-maximum suppression. Afterwards, the feature map and the last remaining ROI are sent to the RoIAlign layer, enabling each ROI to generate a fixed-size feature map. Finally, the flow passes through two branches, one branch enters the fully connected layer for object classification and frame regression, and the other branch enters the full convolutional network (FCN) for pixel segmentation.

### Hb concentration prediction network

CNNs perform well in different types of visual recognition tasks such as image classification (34), target detection (35), and semantic segmentation (36). One of the most important factors leading to the success of CNNs is the intensive training of the availability of images. However, in the medical field, it is difficult to collect sufficient training images with clear labels for Hb content prediction. The difficulty in the collection of a large and accurate training set is attributed to the trouble of providing accurate Hb value labels for eyelid images of patients (even for domain experts) and the time-consuming collection of eyelid images and Hb values of patients, which may limit the number of training samples.

Nonetheless, without the traditional recognition problem, the fluid concentration in the eyelid is in a naturally sequential gradient from 6 to 18 mol/L, and the sequential data among classes will be ignored if labels receive a one-hot encoding. In a given eyelid image of a patient, we are interested in estimating the Hb value of the patient. For two-input eyelid images *X1* and *X2* with ground-truth labels *y1* and *y2*, if there is a close correlation between *y1* and *y2*, the correlation between *X1* and *X2* should be similar, and vice versa. For example, eyelid images with Hb values ranging from 9 to 11 moL/L should have closer correlations than those with Hb values ranging from 9 to 16 moL/L. In other words, close correlations are expected among input images with similar output features. Hence, each instance can be assigned with a discrete label distribution *y* according to its basic facts using label correlation. Label distribution can naturally describe the fuzzy information among all possible labels. Through label distribution learning, the training instances mapping to each class of labels are increased significantly, but the total number of training instances does not actually become larger, which can reduce the training sample space. In this study, the concentration prediction neural network was thus designed based on CNNs and label distribution learning.

Given that *X* represents the input image of the patient's eyelid, *y* is the real label, whose continuous value is *y* ∈ [6, 18]. The model was trained to predict a value as close as possible to the real label. To make use of label correlation, the truth value *y* was transformed into a normal distribution *p*(*y*, σ) to represent a new label. The mean value was set to the value of real label *y*, and σ is the standard deviation of a normal distribution. *Also, p*_*k*_(*y*, σ) is the *k*^−^th element of *p*(*y*, σ), and the continuous value of *k* is *k* ∈ [5, 20]; *p*_*k*_(*y*, σ) can be expressed as follows:


(3) $$\;p_{k}\left(y,\sigma \right)\;=\;\frac{1}{\sqrt{2\pi \sigma }}exp(-\frac{{\left(k-y\right)}^{2}}{2\sigma^{2}})$$

where *p*_*k*_ represents the probability that the truth value of Hb is *k*.

In the training process, assume that *G*(*, θ) is the classification function of the training estimation model, θ is the model parameter, and *z*(*X*, θ) = *G*(*X*, θ). The input image *X* was transformed into a classification vector *z*(*X*, θ), which was then transformed into a probability distribution $\hat{P}\left(X,\theta \right)\;$through the softmax function, and the *k*-th element was expressed as follows:


(4) $$\hat{P}\left(X,\theta \right)\;=\;\frac{exp(z_{k}(X,\theta ))}{\sum_{n}exp(z_{n}(X,\theta ))}$$

where *z*_*k*_(*X*, θ) is the *k*-th element of *z*(*X*, θ).

The difference between the predicted distribution and the real distribution was measured using Kullback–Leibler (K–L) divergence (37). Based on the K–L loss, the model parameters were updated using a stochastic gradient descent (SGD) optimizer, so as to make the two distributions nearly similar.


(5) $$L_{KL}\left(X,y,\theta ,\sigma \right)\;=\;\sum_{k}p_{k}(y,\sigma )ln\frac{p_{k}(y,\sigma )}{{\hat{p}}_{k}(X,\sigma )}$$

After the label distribution learning, the truth value of Hb concentration predicted by the model is shown in Figure 1, and L1Loss was adopted to calculate the loss between the truth value and the predicted value. The overall loss was calculated below:


(6) $$\hat{l}(x)\;=\;L_{KL}\left(X,y,\theta ,\sigma \right)+L1Loss\left(y^{\prime },y\right)$$

where *y*′ is the predicted value, *y* is the truth value.

With the aim of facilitating the subsequent implementation of the concentration prediction function in the mobile terminal, several kinds of lightweight CNNs, including MobileNetv3 (38), MobileNetv2 (39), ShuffleNetv2 (40), SqueezeNet (41), and ResNet CBAM (42) and BCNN (43), were selected as the backbone networks to extract features in this study. Details are shown in the experiments.

## Experimental results and analysis

### Data description

**Figure 4:**
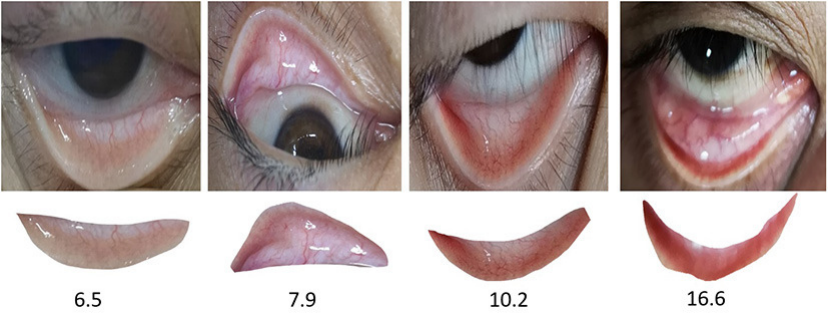
Raw input eye and eyelid image.

**Figure 5:**
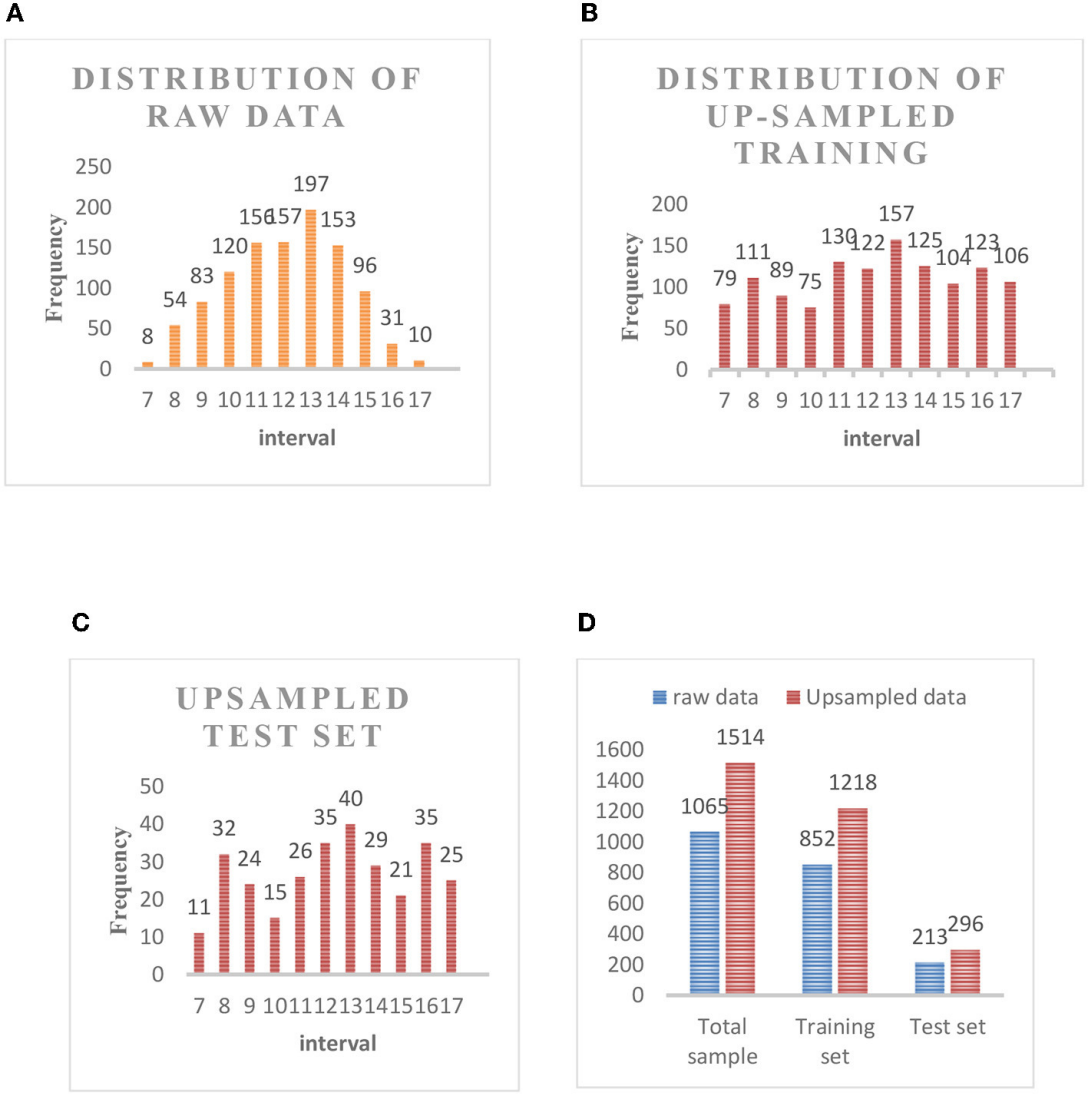
**(A)** Distribution of raw data. **(B)** Distribution of up-sampled training set. **(C)** Up-sampled test set. **(D)** Sample data size.

The images of 1,065 patients receiving surgery were collected from the Department of Anesthesiology at Southwest Hospital as the data set in this experiment. The Hb concentration in all patients was consecutive values distributed between 6 and 18 moL/L (we got the Hb concentration through formal laboratory testing). As shown in Figure 4, the above picture is the original collected eye picture, and the bottom is the manually cropped eyelid picture. The raw data distribution (Figure 5A) showed that the data were imbalanced as there were more data of patients with a normal Hb level but fewer data of the two extremes (patients with a low or high Hb level). The reason is that the images of patients without lesions are common, whereas it is difficult to collect images of patients with lesions or even severe lesions, which also reduces the generalization ability of machine learning algorithms. In this study, therefore, the samples were balanced by up-sampling by synthetic minority oversampling Technology (SMOTE). In detail, the data were divided into the training set and the test set, and then up-sampled separately to balance the data. After up-sampling, the specific distribution of the training set is shown in Figure 5B, that of the test set is shown in Figure 5C, and that of the sample data size is shown in Figure 5D.

### Evaluation indexes

In order to quantitatively evaluate the comprehensive performance of the eyelid image extraction algorithm based on Mask RCNN, this paper adopts Average Recall (AR), Average precision (AP), and mean intersection over union (mIOU) as the evaluation of eyelid image extraction index.

In order to quantitatively evaluate the performance of accurate prediction of hemoglobin concentration, three indexes are used in this study, which are *R*^2^ coefficient, mean absolute error (MAE), and the explained variance score (EVS). Smaller MAE meant better model fitting. Also, r2 is in a theoretical range of [-∞, 1] and a normal range of [0, 1]. The closer of *R*^2^ to 1, the stronger the explanatory ability of the variables of the equation to *y*, and the better the model fitting to data. Besides, the closer of *R*^2^ to 0, the worse the model fitting, and the empirical value >0.4 represented the better fitting effect.

### Eyelid segmentation experiment

These experiments used the open source Pytorch learning framework, Python language programming to realize the algorithm network. In addition, the hardware environment is Dawning workstation from Chongqing Institute of Green and Intelligent Technology, Chinese Academy of Sciences, equipped with dual NVIDIA 2080Ti graphics cards (11 GB), 64-bit Ubuntu16.04 operating system.

In the segmentation experiment, the existing network structure of COCO training set mask-RCNN was directly adopted after the fine-tuning based on the existing weights. A total of 852 eye images was selected for fine-tuning training and 213 images for testing. The training set received multi-scale training, with a batch size of 8. SGD was adopted, and when the learning rate (LR) = 0.01 and num epochs = 24, StepLR decay was employed. The LR declined by 1/2 in the 16th epoch, and it doubled in the 20th epoch. The eyelid segmentation results are shown in Table 1. Figure 4 shows the results of segmentation.

**Table 1:** The average precision and recall under different thresholds of mIoU.

**Method**	**Threshold of IOU**	**Average precision**	**Average recall**	**mIOU**
Mask-RCNN	EYE			
	0.5	0.988	0.991	0.678
	0.5:0.95	0.588	0.660	
	0.75	0.662	0.781	
Swin-transformer	COCO			ADE20K
	0.5	0.709	–	0.535
	0.5:0.95	0.519	–	
	0.75	0.565	–	

**Figure 6:**
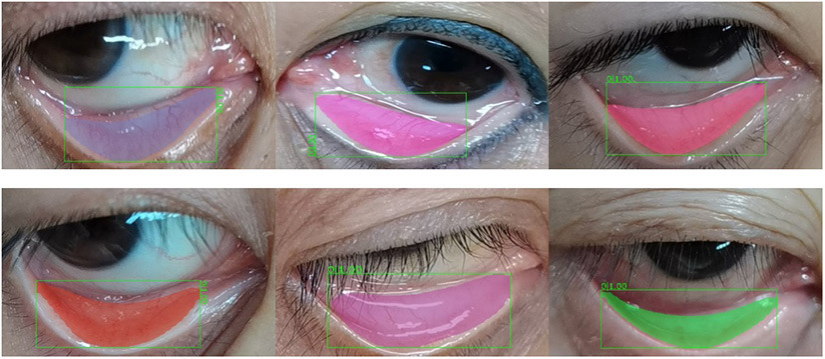
Schematic diagram of segmentation results.

The output result of eyelid semantic segmentation is shown in Figure 6, The effect of eyelid segmentation is very good. The value in the first row in Table 1 indicates that only the prediction boxes with IOU ≥0.5 are counted, and Average Precision and Average Recall of this prediction box are calculated. The second row represents the Average Precision and Average Recall of the prediction boxes under the IOU thresholds that increase from 0.5 to 0.95 with the size of 0.05. Obtain 10 groups of Average Precision and Average Recall, and finally calculate the Average. The value in the third line indicates that only the prediction boxes with an IOU ≥0.75 are counted, and the Average Precision and Average Recall of this prediction box are calculated. mIOU: It refers to the IOU average of all predicted targets and Ground truth.

When the IOU threshold is 0.5, the Average Precision and Average Recall of the model are the largest, which can reach more than 0.98. When the IOU threshold is 0.75, the Average Precision of the model is 0. 662 and Average Recall is 0.781, which is lower than when IOU threshold is 0.5. This is because more strict IOU threshold is used in statistics, which is in line with common sense. When the threshold is 0.5: 0.95, the Average Precision of the model is 0.588, and the Average Recall is 0.660. This is because when the IOU threshold is larger (IOU > 0.75), the conditions for selecting prediction targets are more demanding, which will lead to a further decline in the model results. The Average Precision of the model also reached more than 0.58, which also proved that the model was very ideal. Since we do not have the Average Precision related to eyelid detection as a reference, this paper refers to Swin-Transformer (44), the latest research result in computer vision. Although the data set is different, it can be explained that in the latest research results, the Average Precision of coco data set can reach 0.709 when the IOU threshold is 0.5 (our Average Precision is 0.988), which is a good model. In terms of mIOU, our mIOU is 0.678, which is far higher than Swin-Transformer's index of 0.535 in ADE20K.

### Accurate hemoglobin concentration prediction experiment

In this study, the backbone network for Hb concentration prediction included the fine-grained feature extraction network B-CNN, lightweight convolution image classification networks (MobileNetv3, MobileNetv2, SqueezeNet, and Shufflenetv2), and the residual network combined with attention mechanism (ResNetCBAM). Additionally, an experiment was conducted based on the palpebral conjunctiva feature engineering method (see Supplementary material). In this experiment, the small model parameter of mobilenetv3 was 0.75. The SEblock layer was added to the last layer of backbone networks for feature extraction. Table 2 shows the specific network structure of mobilenetv3. 10-fold cross-validation was performed for all models, and images were cropped to 224 × 224. On the basis of the SGD algorithm, the descent kinetic energy was set to 0.9, the weight decay was 5e-4, the batch size was 64, the LR was initialized to 0.01, and the total epoch was 300. Besides, over-fitting should be avoided using a premature stop strategy.

**Table 2:** Structure of Mobilnetv3+SE.

**Input**	**Operator**	**Exp size**	**#Out**
224 × 224 × 3	conv2d, 3 × 3	–	16
112 × 112 × 16	bneck, 3 × 3	16	16
56 × 56 × 16	bneck, 3 × 3	72	24
28 × 28 × 24	bneck, 3 × 3	88	24
28 × 28 × 24	bneck, 5 × 5	96	40
14 × 14 × 40	bneck, 5 × 5	240	40
14 × 14 × 40	bneck, 5 × 5	240	40
14 × 14 × 40	bneck, 5 × 5	120	48
14 × 14 × 48	bneck, 5 × 5	144	48
14 × 14 × 48	bneck, 5 × 5	288	96
7 × 7 × 96	bneck, 5 × 5	576	96
7 × 7 × 96	bneck,5 × 5	576	96
7 × 7 × 96	conv2d, 1 × 1	–	576
7 × 7 × 576	Pool, 7 × 7	–	–
1 × 1 × 576	conv2d, 1 × 1	–	1,024
1 × 1 × 1,024	AdaptiveAvgPool2d	–	–
1 × 1 × 1,024	conv2d, 1 × 1	–	64
1 × 1 × 64	BatchNorm2d	–	64
1 × 1 × 64	conv2d, 1 × 1	–	64
1 × 1 × 64	ReLU	–	64
1 × 1 × 64	conv2d, 1 × 1	–	15

**Figure 7:**
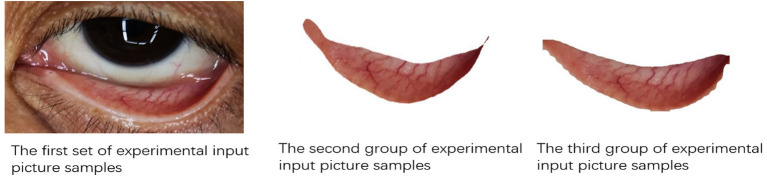
The input images of each group.

Experiment with up-sampled data set (Train: 1,218, Test: 296). The experiment was conducted three times in three groups. First, the Hb concentration was predicted based on the palpebral conjunctiva images artificially selected by the feature engineering method. Secondly, the experiment was based on the original eye images and the feature engineering method. In the end, experiments based on deep convolution network are carried out to verify the model in this paper. The input images of each group are shown in Figure 7. Table 3 shows the experimental results. As revealed by the linear regression model established based on palpebral conjunctiva images artificially selected by the feature engineering method, the optimal effect could be achieved when the coefficient *r*^2^ was 0.3, EVS was 0.304, and MAE was 1.995. The experiment using original eye images based on feature engineering showed that all models failed to predict effectively. The optimal effect could be obtained when random forest *r*^2^ was −0.019, EVS was −0.004, and MAE was −2.421, revealing that the original eye images contained a lot of pseudo-correlation noise that disturbed the prediction of Hb. Hence, based on a priori causal knowledge, it is necessary to locate the palpebral conjunctiva and predict Hb in conjunctiva regions. In this study, the *r*^2^, EVS, and MAE of the model designed based on deep neural networks were superior to those of palpebral conjunctiva images artificially selected by the feature engineering method. The worst model was the Shufflenetv2 network, with the *r*^2^ of 0.321, EVS of 0.357, and MAE of 1.983, while the optimal model was mobilenetv3+SEblock, with the *r*^2^ of 0.512, EVS of 0.535, and MAE of 1.521, which were 0.202, 0.214, and 0.378, respectively, higher than those of images selected based on the feature engineering method. Moreover, comparison in experiments revealed that the prediction efficacy of mobilenetv3+SE was better than all other deep learning models. In comparison with that of the Shufflenetv2 network, the prediction efficacy of mobilenetv3+SE was improved with the *r*^2^ of 0.181, EVS of 0.161, and MAE of 0.366, respectively. It can be concluded that the model based on deep neural networks is superior to the traditional artificial feature engineering method, and the design of the network structure can also affect the prediction of the final Hb concentration.

**Table 3:** Model experimental results with 95% confidence intervals.

**Methods**	***R* ^2^**	**EVS**	**MAE**
**Prediction results of palpebral conjunctiva images artificially selected by feature engineering method**			
Decision tree	0.262 (0.242, 0.283)	0.267 (0.247, 0.287)	2.054 (2.028, 2.080)
Linear regression	0.300 (0.288, 0.312)	0.304 (0.292, 0.315)	1.995 (1.979, 2.010)
SVM	0.267 (0.248, 0.286)	0.270 (0.252, 0.289)	2.042 (2.019,2.064)
K-nearest neighbor regression	0.249 (0.230, 0.268)	0.251 (0.233, 0.27)	2.057 (2.036,2.078)
Random forest regression	0.285 (0.270, 0.299)	0.287 (0.273, 0.302)	2.028 (2.010, 2.047)
Boosting tree regression	0.296 (0.283, 0.308)	0.298 (0.287, 0.31)	2.012 (1.995,2.029)
**Prediction results of original eye images base on feature engineering**			
Decision tree	−0.013 (−0.032, 0.006)	−0.001 (−0.021, 0.018)	2.425 (2.404,2.447)
Linear regression	−0.077 (−0.113, −0.041)	−0.064 (−0.101, 0.027)	2.462 (2.427,2.496)
SVM	−0.052 (−0.081, −0.024)	−0.039 (−0.068, −0.01)	2.428 (2.401,2.455)
K-nearest neighbor regression	−0.021 (−0.040, −0.001)	−0.006 (−0.026, 0.014)	2.401 (2.382, 2.420)
Random forest regression	−0.019 (−0.041, 0.003)	−0.004 (−0.027, 0.019)	2.421 (2.397,2.444)
Boosting tree regression	−0.053 (−0.083, −0.024)	−0.037 (−0.067, 0.007)	2.442 (2.410, 2.473)
**Prediction results of deep CNNs based on a priori causal knowledge**			
BCNN	0.447 (0.446, 0.447)	0.452 (0.451, 0.453)	1.812 (1.812, 1.813)
mobilev2	0.447 (0.445,0.450)	0.462 (0.459, 0.466)	1.822 (1.819, 1.826)
Shufflenetv2	0.321 (0.319,0.323)	0.357 (0.352, 0.364)	1.983 (1.976, 1.991)
Squeezenet	0.498 (0.495,0.502)	0.511 (0.508, 0.514)	1.688 (1.685, 1.693)
Resnet_cbam	0.463 (0.461,0.466)	0.463 (0.461,0.466)	1.719 (1.714, 1.725)
mobilenetv3+SE	0.512 (0.499,0.517)	0.535(0.515, 0.542)	1.521 (1.481, 1.574)

### Ablation experiment

Ablation experiments were conducted to validate the influence of eyelid segmentation on the mobilenetv3+SE neural network that is the optimal model based on experiments. The concentration was predicted by experiments based on original eye images, eyelid images that were artificially cropped, and eyelid images receiving semantic segmentation, respectively. The experimental results (Table 4) illustrated that the *r*^2^ was 0.512, EVS was 0.535, and MAE was 1.520 for eyelid images receiving semantic segmentation, 0.503, 0.518, and 1.528 for artificially cropped eyelid images, and 0.306, 0.338, and 1.952 for original eye images, respectively. It seems that eyelid region extraction by causal knowledge is beneficial to improving the prediction efficiency of the Hb concentration and identifying the effectiveness of the method proposed in this study.

**Table 4:** Ablation experiment results with 95% confidence intervals.

**Methods**	***R* ^2^**	**EVS**	**MAE**
**Eyelid images receiving semantic segmentation**			
mobilenetv3+SE	0.512 (0.505, 0.512)	0.535 (0.513, 0.519)	1.520 (1.515, 1.526)
**Artificially cropped eyelid images**			
mobilenetv3+SE	0.503 (0.499, 0.507)	0.518 (0.515, 0.522)	1.528 (1.511, 1.574)
**Original eye images**			
mobilenetv3+SE	0.306 (0.296, 0.317)	0.338 (0.329, 0.348)	1.952 (1.936, 1.967)

## Conclusion and prospect

Hb detection is the foremost part of the diagnosis of perioperative anemia, and it is expected to be rapid, non-invasive, real-time, and easy to operate under the ideal condition. As artificial intelligence technology advances, computer vision has been widely applied in image recognition and analysis in the medical field. The recognition and analysis of palpebral conjunctiva images by machine learning not only has higher accuracy than that by doctors' visual method but also can make up for the shortcomings caused by the lack of medical personnel as well as the deficiency and backwardness of medical equipment. Besides, patients with anemia receive blood transfusion in light of their Hb levels, so they need to be monitored frequently. Thus, their Hb levels are usually measured invasively. Invasive methods are generally not recommended, especially for infants, the elderly, pregnant women, patients with anemia, and those with sickle cell disease. Frequent blood sampling will make patients feel extremely uncomfortable, and its cost is quite high, especially in areas with limited economic resources worldwide. It is important to research methods and design tools to monitor Hb concentration in a non-invasive way to reduce the cost of patients. In this study, we proposed to predict the accurate hemoglobin concentration and finally constructed a model using the deep learning method to predict eyelid Hb of perioperative patients based on the a priori casual knowledge. The model is effective and practical through verification by experiments of the real medical data set, of which the *R*^2^ can reach 0.512, the explained variance score can reach 0.535, and the mean absolute error is 1.521. For the shortcomings of this study and the future work direction, firstly, there are too few data sets, and the generalization ability of the model needs to be improved, so extended research should be conducted based on more eyelid pictures collected from cooperative hospitals in the future. Secondly, the model parameters of the eyelid image segmentation algorithm based on mask-RCNN are too large to be transplanted to the mobile terminal, therefore, in the future, a more compact model should be designed to segment eyelid images. Additionally, developing a model based on the deep learning method into a practical system is also the exploration field of this study in the future.

## Data availability statement

The original contributions presented in the study are included in the article/Supplementary material, further inquiries can be directed to the corresponding author.

## Ethics statement

The studies involving human participants were reviewed and approved by the Institutional Ethics Committee of the First Affiliated Hospital of Third Military Medical University (also called Army Medical University) (No. KY2021060). The patients/participants provided their written informed consent to participate in this study.

## Author contributions

Study concept and design: YC and YZ. Analysis and interpretation of data: YZ and KZ. Technical support: YC and QS. Obtained funding: YC. Writing–original manuscript and revision of manuscript: YC and KZ. All authors contributed to the article and approved the submitted version.

## Funding

The work was supported by Chongqing Municipal Natural Science Foundation (Number: 2022NSCQ-MSX0241).

## Conflict of interest

The authors declare that the research was conducted in the absence of any commercial or financial relationships that could be construed as a potential conflict of interest.

## Publisher's note

All claims expressed in this article are solely those of the authors and do not necessarily represent those of their affiliated organizations, or those of the publisher, the editors and the reviewers. Any product that may be evaluated in this article, or claim that may be made by its manufacturer, is not guaranteed or endorsed by the publisher.
