# Supplementary material for: Two-stage hemoglobin prediction based on prior causality
Source: Front Public Health. 2022 Nov 30;10:1079389. doi: 10.3389/fpubh.2022.1079389 (PMC9748421; doi:10.3389/fpubh.2022.1079389)
Supplement: Supplementary file 1 [file Table_1.docx]

Supplementary Material

# Hb Prediction by Eyelid Images Based on Feature Engineering

Hb concentration is related to eyelid color, so a feature engineering method was designed for experiments, mainly involving such features as the color moment of images, the theme color difference of images, and 13 features proposed in the literature[1]. In the present study, these features were fused as feature vectors of eyelid images, and experiments were carried out on several regression models (decision regression tree, linear regression, SVM, k-nearest neighbor regression, random forest regression, and boosting tree regression).

# Color Moments

Image features are a set of different attributes that can characterize the features or contents of images, mainly including natural features of images (such as brightness, color, and texture) and man-made features of images (e.g., image spectrum, image histogram). Moments are crucial statistics that characterize data distribution. In statistics, the first moment refers to the mean value of data distribution, the second moment represents the variance of data distribution, and the third moment stands for the skewedness of data distribution. The color moment of an image characterizes color distribution in the image is a relatively vital global image feature representation. During the extraction of color moments, the first, second, and third moments were the main targets, which can manifest the color distribution of digital images. The extraction of color moments requiring no pre-quantification of color features is superior to that of color histograms.

For digital image P, the first color moment is calculated using the following formula:

$\boldsymbol{\mu}_{\boldsymbol{i}}\boldsymbol{=}\frac{\boldsymbol{1}}{\boldsymbol{N}}\sum_{\boldsymbol{j = 1}}^{\boldsymbol{N}} \boldsymbol{P}_{\boldsymbol{ij}}$,

where$P_{ij}$ is the value of the j-th pixel of the digital image P at the i-th color channel, and N is the number of pixels in the image.

The second color moment is calculated using the following formula:

$$\boldsymbol{\sigma}_{\boldsymbol{i}}\boldsymbol{=}\left[ \frac{\boldsymbol{1}}{\boldsymbol{N-1}}\sum_{\boldsymbol{j = 1}}^{\boldsymbol{N}} {\boldsymbol{(}\boldsymbol{P}_{\boldsymbol{ij}}\boldsymbol{-}\boldsymbol{\mu}_{\boldsymbol{i}}\boldsymbol{)}}^{\boldsymbol{2}} \right]^{\frac{\boldsymbol{1}}{\boldsymbol{2}}}$$

The third color moment is calculated using the following formula:

$$\boldsymbol{s}_{\boldsymbol{i}}\boldsymbol{=}\left[ \frac{\boldsymbol{1}}{\boldsymbol{N}}\sum_{\boldsymbol{j = 1}}^{\boldsymbol{N}} {\boldsymbol{(}\boldsymbol{P}_{\boldsymbol{ij}}\boldsymbol{-}\boldsymbol{\mu}_{\boldsymbol{i}}\boldsymbol{)}}^{\boldsymbol{3}} \right]^{\frac{\boldsymbol{1}}{\boldsymbol{3}}}$$

The first moment can be interpreted as the average response intensity at the color channel, the second moment is the response variance at the color channel, and the third moment can characterize the skewedness of the data distribution at the color channel. For a color image, there are none color moments, with three low-order moments at each color channel.

# Theme Color Extraction

Color quantization is the process of reducing the number of colors in an image through merging the less important similar colors in original images into one color by virtue of the inertia of human eyes to color while minimizing the cognitive error of the image before and after quantization for human eyes, namely, the smallest quantization error. Color quantization is a basic technology for digital image processing. Traditional quantization algorithms include segmentation and clustering. In this study, the red space in eyelid images was quantified as the feature of the images, and the theme color was extracted using the k-means clustering method. As a result, two theme colors were extracted, and the Euclidean distance between them was taken as the feature value.

Supplementary Figure 1 shows the color distribution of eyelid images in RGB color space, and Supplementary Figures 2 and 3 display the schematic diagram of color palettes for extracting 10 and 2 colors.


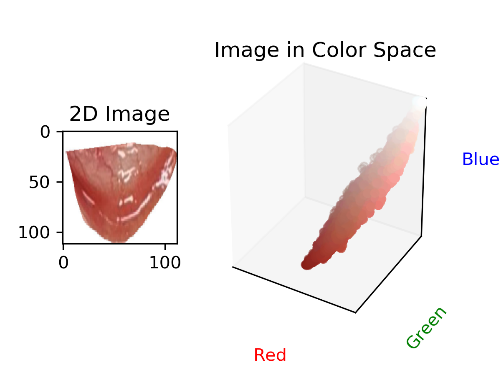


**Supplementary Figure 1.** Color distribution of eyelid images in RGB


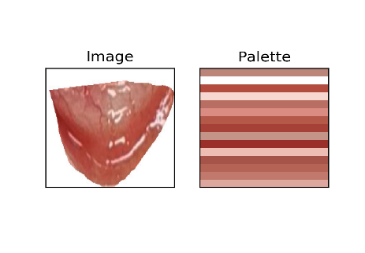


**Supplementary Figure 2.** Theme color palette for 10 colors


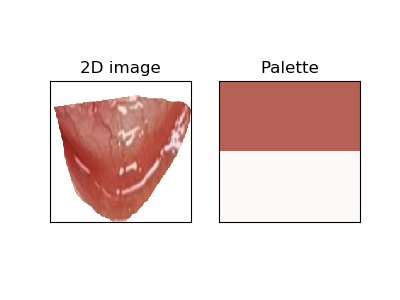


**Supplementary Figure 3.** Theme color palette for 2 colors

The theme color of each color is:

Theme_color_1_R = Tc1_R

Theme_color_1_G = Tc1_G

Theme_color_1_B = Tc1_B

Theme_color_2_R = Tc2_R

Theme_color_2_G = Tc2_G

Theme_color_2_B = Tc2_B

The final theme color features were calculated by the following formula:

$$\boldsymbol{M =}\sqrt[\boldsymbol{2}]{{\boldsymbol{(Tc}\boldsymbol{1\_R}\boldsymbol{-}\boldsymbol{Tc}\boldsymbol{2\_R)}}^{\boldsymbol{2}}\boldsymbol{+}{\boldsymbol{(Tc}\boldsymbol{1\_G}\boldsymbol{-}\boldsymbol{Tc}\boldsymbol{2\_G)}}^{\boldsymbol{2}}\boldsymbol{+}{\boldsymbol{(Tc}\boldsymbol{1\_B}\boldsymbol{-}\boldsymbol{Tc}\boldsymbol{2\_B)}}^{\boldsymbol{2}}}$$

With reference to 13 features in the literature[1], the remaining feature vectors in feature engineering were finally fused into feature vectors of 23 dimensions, and the Hb was predicted and evaluated by traditional regressors.

**Reference**

1. Chen YM, Miaou SG, Bian HJCM, Biomedicine Pi. Examining palpebral conjunctiva for anemia assessment with image processing methods. 2016:125-35.
